# Fifteen Years after the Definition of *Trypanosoma cruzi* DTUs: What Have We Learned?

**DOI:** 10.3390/life13122339

**Published:** 2023-12-14

**Authors:** Bianca Zingales, Andréa M. Macedo

**Affiliations:** 1 Departamento de Bioquímica, Instituto de Química, Universidade de São Paulo, São Paulo 05508-900, São Paulo, Brazil; 2 Departamento de Bioquímica e Imunologia, Instituto de Ciências Biológicas, Universidade Federal de Minas Gerais, Belo Horizonte 31270-901, Minas Gerais, Brazil; andrea@icb.ufmg.br

**Keywords:** *Trypanosoma cruzi*, discrete typing units, genomics, Chagas disease manifestations, ecoepidemiology

## Abstract

*Trypanosoma cruzi*, the protozoan causative of Chagas disease (ChD), exhibits striking genetic and phenotypic intraspecific diversity, along with ecoepidemiological complexity. Human-pathogen interactions lead to distinct clinical presentations of ChD. In 2009, an international consensus classified *T. cruzi* strains into six discrete typing units (DTUs), TcI to TcVI, later including TcBat, and proposed reproducible genotyping schemes for DTU identification. This article aims to review the impact of classifying *T. cruzi* strains into DTUs on our understanding of biological, ecoepidemiological, and pathogenic aspects of *T. cruzi*. We will explore the likely origin of DTUs and the intrinsic characteristics of each group of strains concerning genome organization, genomics, and susceptibility to drugs used in ChD treatment. We will also provide an overview of the association of DTUs with mammalian reservoirs, and summarize the geographic distribution, and the clinical implications, of prevalent specific DTUs in ChD patients. Throughout this review, we will emphasize the crucial roles of both parasite and human genetics in defining ChD pathogenesis and chemotherapy outcome.

## 1. Introduction

The hemoflagellate protozoan *Trypanosoma cruzi* is the causative agent of Chagas disease, also known as American Trypanosomiasis, that affects 6–7 million people worldwide, with about 8000 deaths every year [1]. Although the greatest health burden of Chagas disease is still in Latin America, due to population mobility the infection has increasingly been detected in the United States, Canada, and many European countries [1].

The parasite *T. cruzi* is mainly transmitted to humans by contact with feces/urine of infected blood-sucking triatomine insects, but blood transfusion, oral and congenital transmission routes also play important roles [2]. There is no available vaccine for Chagas disease and the treatment mainly relies on the nitroheterocyclic compounds Benznidazole (BZN) and Nifurtimox (NFX), which have been in use for over half a century, face safety issues, and variable efficacy according to the phase of the disease [3,4]. While the drugs prove effective in treating the acute phase and early infection in children, therapeutic failures are common in the chronic phase in which most patients are diagnosed [3].

Human infection begins with an acute phase, in which most individuals, despite having patent parasitemia, are asymptomatic. After the acute phase, which can last up to three months, the infection progresses to a chronic phase, characterized by subpatent parasitemia and, in the great majority of individuals, an absence of detectable symptoms (indeterminate clinical form). However, at least 30% of asymptomatic individuals develop, in the following years, a predominantly inflammatory response, which leads to potentially lethal symptomatic forms, including digestive and cardiac manifestations [5]. The complex network of events that underlie the generation of protective versus pathogenic immune responses during the chronic phase and the progression to cardiomyopathy are not completely understood [5,6]. However, evidence indicates that the host immune response and the heterogeneity of parasite strains play important roles [5].

The diversity of *T. cruzi* strains and the multiplicity of their genotypes and phenotypes have long been recognized along with their ecoepidemiological complexity, topics widely reviewed elsewhere [7,8,9]. *T. cruzi* is predominantly diploid, divides by binary fission [10] and was, for a long time, accepted as having basically a clonal population structure [11]. In this clonal model, new genotypes were thought to evolve through the gradual accumulation of discrete mutations, with little influence from rare events of genetic recombination [11]. However, emerging evidence has challenged this traditional perspective. Studies identified an excess of heterozygosity in individual genes in natural populations of *T. cruzi* [12,13,14,15,16,17]. Furthermore, the documentation of genetic exchange in vitro [18,19], coupled with compelling evidence suggesting that sexual reproduction occurs in nature and that clonal and sexually panmictic populations can coexist [20,21], strongly support the presence of a sexual cycle in *T. cruzi*. The hybrid genotypes, later stabilized by clonal propagation, must have played a substantial role in shaping the genetic diversity of this parasite.

In 2009, an expert committee considering the genetic diversity of *T. cruzi* strains and previous attempts to cluster them into relevant subgroups, based on genetic, biochemical or biological markers, recommended that the parasite strains should be assigned to one of six discrete typing units (DTUs), termed TcI to TcVI [22]. Subsequently, a seventh DTU, initially restricted to bat species [23] and named TcBat, was included [24,25]. The term DTU refers to discrete evolutionary lineages, identifiable by common molecular markers, wherein the parasites belonging to one group are genetically more similar to each other than to any other group [26]. However, even though phylogenetically more related to each other, *T. cruzi* populations within a DTU are not genetically identical, as these parasites accumulate mutations, experience varying degrees of genetic exchange and undergo clonal propagation, which allows differentiating them with additional markers [27].

The correspondence between the DTUs and former nomenclatures of *T. cruzi* groups can be found elsewhere [7,22,28,29], and will not be further discussed in this review. The purpose of categorizing *T. cruzi* strains into a unified nomenclature was to facilitate communication within the scientific community involved in *T. cruzi* and Chagas disease research [22]. To obtain the expected results, simple and reproducible schemes, based on the amplification of a set of nuclear DNA sequences, were proposed for typing isolates into their respective DTUs [24]. This approach, widely used in endemic areas of Chagas disease, has guided the study of the biological aspects and ecoepidemiological and pathogenic characteristics of *T. cruzi*. It is important to mention that although there is substantial intra-DTU genetic diversity, in most studies genotyping is performed only at the lineage level.

The compilation of data published in 137 articles until April 2016 describes the geographic distribution of 6400 DTUs found in human, vector, and mammalian reservoirs in Chagas disease endemic areas [30]. In the present review, these data will be updated.

The aim of this article is to analyze the impact that the definition of the seven DTUs has had on the knowledge of *T. cruzi* biology and characteristics of each group of strains regarding genome organization and susceptibility to drugs. We will also review documentation on the association of the DTUs with mammalian reservoirs found in the *T. cruzi* sylvatic cycle. In the context of Chagas disease, we will review the geographic distribution of DTUs prevalent in patients and evidence of the potential association of certain DTUs with clinical manifestations.

## 2. Origin of *T. cruzi* DTUs

Based on the analysis of nuclear markers, some models have been proposed to describe the origin and relationships between the DTUs [24,29,31,32,33]. The most widely accepted hypothesis is that TcI and TcII represent ancestral lineages that have been evolving independently for a long time. It is also extensively accepted that TcV and TcVI have a hybrid origin, with TcII and TcIII considered as their parental lineages. However, it is unclear whether TcV and TcVI are progeny from a single hybridization event, followed by clonal diversification [31] or from two independent hybridization events [32,34,35]. In any case, the events that led to the emergence of the hybrid lineages TcV and TcVI appear to be evolutionarily ancient and, according to the method used for dating, were estimated to have occurred less than 1 million years ago (mya) [36] or within the last 60,000 years [35].

The evolutionary origins of DTUs TcIII and TcIV have been a matter of debate. Some authors suggested that these DTUs could also be hybrids [29,31]. Other authors, however, have proposed that TcIII and TcIV are pure lineages that evolved from a common ancestor of TcI [32,34]. Multilocus phylogenetic analyses of nuclear genes place Tcbat clade closer to TcI [25].

The population structure of *T. cruzi* was also analyzed from genealogies of mitochondrial sequences and three independent clades were disclosed, which hold a good correlation with the DTUs: mtTcI, corresponds to TcI; mtTcII, to TcII; and mtTcIII encompasses the TcIII, TcIV, TcV, and TcVI strains [32,37]. A comprehensive study characterized *T. cruzi* mtTcI and mtTcIII lineages circulating in North and Central America and concluded that mtTcIII of North and Central America represents a distinct clade from South America [38].

## 3. Intrinsic Characteristics of the DTUs

In this review, when we mention the intrinsic characteristics of the DTUs, we are referring to properties that are inherent to the strains of a given DTU and that have been determined experimentally. In addition, to facilitate comprehension of the upcoming text, we summarize the developmental stages found in the *T. cruzi* life cycle and those used in in vitro studies.

In the classic version, the life cycle of *T. cruzi* comprises three main developmental forms: the epimastigote, which proliferates in the gut of triatomine vectors; the trypomastigote, a non-dividing form capable of invading mammalian cells; and the amastigote, which multiplies in the cytoplasm of infected cells. Notably, trypomastigotes found in the blood of the mammalian host and trypomastigotes present in the excreta of the blood-feeding triatomine vectors, known as metacyclic trypomastigotes, exhibit considerable differences, mainly in their cell surface composition [39].

An updated view of the *T. cruzi* life cycle incorporates intracellular epimastigote-like forms in the mammalian host [40]. These forms could represent an intermediate stage in the transition from amastigotes to trypomastigotes [41]. Further, observations of in vitro and in vivo *T. cruzi* infections recognized a population of non-proliferating intracellular amastigotes that were able to spontaneously differentiate into trypomastigotes and propagate the infection [42]. This phenomenon, defined by the authors as “spontaneous dormancy”, suggests an inherent capacity of amastigotes to tolerate changing environments. In fact, *T. cruzi* amastigotes reduce their replication rate as a response to stress, including metabolic blockades, nutrient starvation, and sublethal exposure to the drug BZN [43]. Such observations may have important implications for understanding parasite persistence in the chronic phase of Chagas disease and for interpreting drug efficacy (see below). In line with these expectations, highly sensitive imaging, at single-cell resolution, revealed that in chronic murine infections the proportion of intracellular parasites in the S-phase is significantly lower than it is during the acute stage [44]. Remarkably, in vitro infections showed that the percentage of “dormant” amastigotes of strains belonging to hybrid DTUs TcV and TcVI was higher when compared to non-hybrid DTU strains TcI and TcII [45]. Depending on the research objectives, various parasite stages are utilized in in vitro assays, including, epimastigotes grown in axenic medium; trypomastigotes obtained from the supernatant of mammalian cultured cells; metacyclic trypomastigotes derived from epimastigote differentiation; and intracellular amastigotes [46]. In these studies, both polyclonal strains and isolated clones have been used. It is worth noting that the available data primarily relate to the strains of DTUs TcI, TcII, TcV, and TcVI, as they are prevalent in human hosts, as discussed below.

### 3.1. Genome and Genomics

Trypanosomatids have two well-characterized genomes: the nuclear genome and the genome found in the unique mitochondrion called kinetoplast. The kinetoplast genome (kDNA) consists of a network of two types of circular DNA molecules, the maxicircles and the minicircles [47]. Maxicircles are equivalent to the mitochondrial DNA of eukaryotes, while the minicircles encode guide RNAs (gRNAs) that act in the process of posttranscriptional modifications. This section will primarily focus on the nuclear genome.

#### 3.1.1. DTU Nuclear Genomes

In the eighties, pioneering studies led by James Dvorak’s group revealed differences of up to 40% in the DNA content of clones of several strains, with nuclear DNA being the main factor responsible for the variability [48]. Later, DNA diversity was assessed in a comprehensive panel of clones within the context of DTU divisions [49]. The overall findings indicated large genome size variations both between and within DTUs. TcI isolates exhibited, on average, the smallest nuclear genomes, and strains of the hybrid DTUs TcV and TcVI had the lowest intragroup variability [49]. The reduced size of the TcI genome was confirmed in subsequent research [50].

In *T. cruzi*, mitosis occurs without a complete disruption of the nuclear envelope and the parasite chromosomes fail to condense during cell division [10]. These characteristics precluded classical cytogenetic studies for the analysis of *T. cruzi* karyotypes. Instead, pulsed field gel electrophoresis (PFGE) in combination with Southern blot have been used. Several studies have demonstrated karyotype variability among parasite strains, with differences of up to 50% in the sizes of homologous chromosomes in many strains (see references cited in [50]).

**Figure 1:**
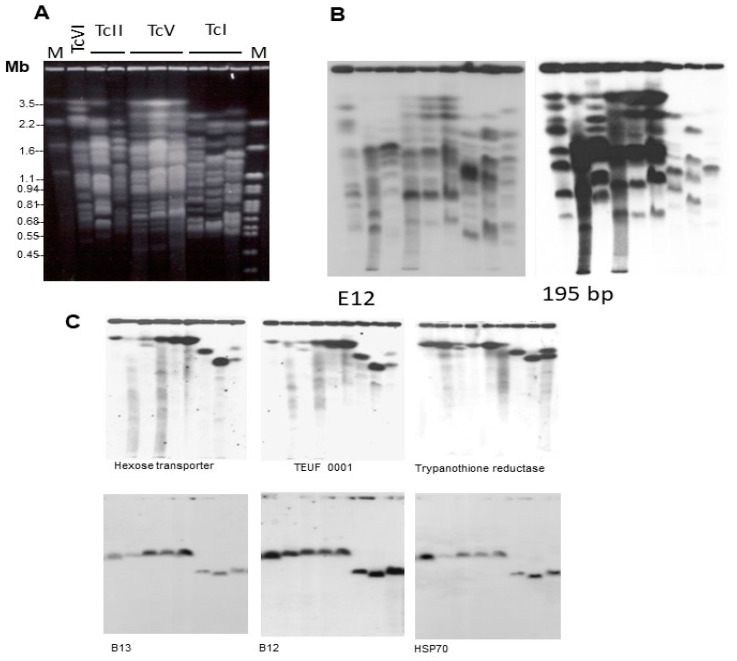
Molecular karyotype of *T. cruzi* strains representative of four DTUs: TcVI (CL Brener), TcII (Esmeraldo cl3 and Y), TcV (NRcl3, SO3 cl5, and Sc43 cl1), and TcI (Silvio X10 cl1, Dm28c and YuYu). (**A**) Chromosomal DNA was separated by PFGE and stained with ethidium bromide. After transfer to Zeta-Probe membranes, the blot was hybridized with 32P-labeled probes corresponding to (**B**) DNA repetitive sequences E12 and 195-bp satellite DNA; and (**C**) single copy genes B13, B12, and HSP70. See experimental details in [51]. M = Molecular size markers. Reprinted/adapted with permission from Ref. [51]. 2004, Elsevier.

To illustrate the conclusions that follow, we compiled Figure 1, derived from previous studies [51].

In Figure 1A, it is evident that the chromosome sizes of TcI strains are generally smaller than those of the other DTUs, providing further support for the conclusion that TcI strains have smaller genomes. Also remarkable is the conservation of the karyotype of three DTU TcV clones, derived from strains of different hosts [51]. As will be discussed later, differences in genome size can be attributable to the amplification and deletion of various repeated DNA sequences. Figure 1B shows the hybridization pattern of the chromosomal bands of the strains, indicated in Figure 1A, with probes corresponding to the E12 repeated interspersed DNA sequence (1123 bp; 5600 copies in the Maracay strain) and the 195-bp satellite DNA (120,000 copies in the Y strain) [52]. These sequences hybridize with many chromosomal bands in all stocks. As expected, the hybridization intensity of the 195-bp probe is higher than the E12 probe. Furthermore, it is notably higher in the TcII, TcV, and TcVI strains in comparison to TcI (Figure 1B). Quantification of the hybridization signal indicated that the 195-bp DNA in CL Brener (TcVI) is approximately seven times more abundant than in TcI strains (Silvio X10 cl1 and Dm28c) [51]. It is important to mention that the 195-bp DNA target has been used for high-sensitivity *T. cruzi* detection [53].

Despite variations in genome size and chromosomal polymorphisms, *T. cruzi* DTUs exhibit conservation of several syntenic groups [50,51,54]. Figure 1C provides an example of two syntenic groups, which are retained in the chromosomes of strains of different DTUs. The preservation of these large synthetic regions suggests that their maintenance is a result of positive selection.

#### 3.1.2. DTU Genomics

In April 1994, the *Trypanosoma cruzi* Genome Project was launched at the Oswaldo Cruz Foundation in Rio de Janeiro, and the CL Brener clone, which had been isolated from the CL strain, was chosen for sequencing. It is worth noting that, at that time, researchers were unaware that the CL strain, and therefore CL Brener, were hybrid strains, which significantly complicated the genome sequencing and assembly. It later became clear that CL and CL Brener belong to the DTU TcVI and originated from the hybridization of TcII and TcIII ancestors [31].

The article describing the Genome Sequence of *Trypanosoma cruzi* was published in *Science* on 15 July 2005 [55], alongside the genome sequencing of the two related pathogens *Trypanosoma brucei* [56] and *Leishmania major* [57]. The comparison of the gene content and genome architecture of the three organisms disclosed a conserved core proteome of about 6200 genes distributed in large syntenic polycistronic gene clusters, alongside substantial genomic differences, which most likely reflect specific adaptations for each organism’s survival and the distinct pathophysiologies they promote [58].

The CL Brener sequencing data confirmed that over 50% of the *T. cruzi* genome consists of repeated sequences, mainly transposable elements, tandem repeats, and multigenic families encoding surface proteins: trans-sialidases (TS), mucins (TcMUC), mucin-associated surface proteins (MASP), and glycoprotein 63 (Gp63), each with hundreds to thousands of members [55]. Further details regarding the characteristics of each of these multigene families and their role in cell invasion, immune evasion, and survival in different hosts can be found elsewhere [9,59,60]. Due to the heterozygous nature of the CL Brener genome, the high content of repetitive sequences, and the sequencing methodology available at that time, the sequence assembly of CL Brener resulted in a fragmented genome with extensively collapsed high-repeat regions [55].

The development of new sequencing technologies, such as short-Ilumina and long-PacBio and -Nanopore reads, has significantly improved the quality of the assembled genomes. As a result, several genomes of strains belonging to different DTUs are now available (see references in [9,61]). The genomic landscape has revealed that *T. cruzi* genes are organized into two compartments, known as “core” and “disruptive” [60]. The “core compartment” consists of conserved and hypothetical conserved genes, presenting the same gene order found in the genomes of *Leishmania* and *T. brucei*. In contrast, the “disruptive compartment” is constituted by rapidly evolving multigene families, which mainly encode polymorphic surface antigens. Phylogenetic trees constructed from whole genome alignments of thirteen strains representing the six DTUs, showed well-supported clades corresponding to the DTUs, confirming that *T. cruzi* DTU genetic structure is present at the genome level [62]. The topology of the tree corroborates the phylogenetic relationships between the DTUs reported by many authors from the analysis of gene or protein sequences and concatenated gene sequences [25].

Within the “disruptive compartment” the surface gene families are highly expanded in the genome of all strains, with a variable number of copies, ranging from 400 to 3200. These copies are clustered in large arrays and distributed along several chromosomes. The abundant concentration of retrotransposons within these clusters suggests that retrotransoposons are involved in recombination processes responsible for generating antigenic diversity [63]. Furthermore, the use of combined long-read sequencing and high-quality assemblies of the genomes of the TcI and TcII strains, representing the putative ancestral lineages of *T. cruzi*, has revealed significant diversity among these isolates. This study also provided additional evidence regarding the processes that generate such diversity, which include gene amplification, dispersion of gene copies throughout the genome, recombination events, and in situ mutations [64].

To study the sequence variability and copy number variation of the TS, MASP, and TcMUC families, a new methodology was developed [65]. The analysis of 36 strains belonging to six DTUs showed that the overall copy number of the three multigene families was lower in TcI and TcII as compared to TcIII-TcVI. When assessing the sequence variability of these multigene families among strains, the isolates of TcI and TcII DTUs formed distinct groups, suggesting the occurrence of DTU-specific motifs, probably caused by their long evolutive divergence. Strains belonging to the hybrid DTUs TcV and TcVI exhibited statistically significant higher variability in multigene families compared to non-hybrid isolates and were more closely related to the TcIII and TcII strains, their parental DTUs [65]. Furthermore, these studies have also highlighted that the mammalian immune response primarily targets the TS multigene family. The substantial expansion and polymorphisms observed in the surface protein genes most certainly have enabled the parasite to establish infection in different hosts and cell types. On the other hand, this variability must have also provided the parasite with a mechanism to evade the host’s immune response, thereby making the development of effective protective vaccines against *T. cruzi* a huge challenge.

### 3.2. In Vitro Susceptibility to Drugs

The current treatment for Chagas disease is restricted to two nitroheterocyclic compounds: the 5-nitrofuran NFX and the 2-nitroimidazole BZN. These agents are prodrugs that require activation of the nitro group by a parasite’s nitroreductase (TcNTR) to exert cytotoxic effects [66,67]. Although extensively studied, the mechanisms of action of both drugs are not yet fully understood. NFX is thought to increase oxidative stress by producing highly toxic reactive oxygen species and free radicals, whereas BZN would lead to the formation of reductive metabolites that can cause deleterious effects, including DNA damage. Discussing the evidence for the proposed mechanisms of action of these drugs is beyond the scope of this review. Instead, our focus will be on analyzing the variations in parasite susceptibility among the different DTUs.

The first observations regarding differences in the susceptibility of *T. cruzi* strains to BZN and NFX came from studies conducted in murine models [68,69,70]. Based on the percentages of cure achieved by the drugs in albino infected mice, the strains were categorized as either drug-sensitive or naturally drug-resistant [68]. Strains whose percentage of cure was less than 50% were arbitrarily considered “naturally drug-resistant”. In this model, Colombiana and YuYu (both of the DTU TcI) emerged as classic representatives of strains highly resistant to BZN and NFX [68]. It is important to clarify that the expression “natural drug resistance” should be understood as indicating an “intrinsic/innate drug resistance” to distinguish it from “acquired drug resistance”, that can be obtained in the laboratory by selection under drug pressure. However, based on the original definition of “naturally drug-resistant strains” by Filardi and Brener [68] it is worth noting that none of the *T. cruzi* stocks are 100% resistant to the nitroheterocyclic drugs.

The observation that *T. cruzi* strains exhibited varying responses to NFX and BZN in the same host and under identical experimental conditions, combined with reports of variability in cure rates with these drugs in Chagas disease patients (as will be discussed below), raised the possibility of natural drug resistance as a potential explanation for treatment failures. This encouraged researchers to explore drug susceptibility in vitro in different isolates, categorized into distinct groups (zymodemes, biodemes, lineages, or DTUs). The first studies focused mainly on evaluating the drug’s activity against epimastigote forms due to its relative simplicity of execution. More recently, researchers have evaluated the effect of compounds against intracellular amastigotes and trypomastigotes. It is worth mentioning that the analysis of the parasiticidal activity of compounds on trypomastigotes presents particularities, as these forms neither divide nor have a long lifespan in vitro.

Different methodologies have been employed to assess drug susceptibility. For epimastigotes and trypomastigotes, parasite viability was primarily determined via microscopic counting. Three main methods for measuring the number of amastigotes per infected cell were used: high-content imaging analysis, microscopic counting after Giemsa staining, and the colorimetric method using the β-galactosidase-transfected Tulahuen strain [71]. In most of the studies, the IC_50_ value was reported, which refers to the concentration of the drug capable of inhibiting 50% of parasite proliferation. LC_50_ (lethal concentration 50), instead of IC_50_, was determined for the non-replicative trypomastigotes. In all cases, it should be emphasized that the absolute values of IC_50_ or LC_50_ are related to the experimental conditions, which include the initial parasite concentration, time of exposure to the drug and the methodology used to measure the results. In this way, only intra-assay comparisons should be considered.

Some early reports suggested that strains of DTU TcI would be more resistant to BZN than strains of other DTUs [72]. However, later studies, with larger panels of strains, mainly of the DTUs TcI, TcII, and TcV, verified a lack of correlation between the in vitro susceptibility to BZN and the phylogenetic diversity of *T. cruzi* [73,74,75,76]. In addition, a high range of intra-DTU sensitivity to BZN was observed [75,77].

Using a high-throughput screening platform, Moraes and co-workers demonstrated that BZN and NFX exhibited broad efficacy against intracellular amastigotes of a panel of *T. cruzi* strains representative of the six DTUs [75]. A surprisingly different pattern of *T. cruzi* response was observed for the triazoles posaconazole (POSA) and ravuconazole, inhibitors of the sterol 14 alpha-demethylase (CYP51) enzyme of the ergosterol biosynthesis pathway. These compounds showed variable activity against amastigotes, which was both compound and strain specific. Noticeably, the 92-80 cl2 strain, belonging to DTU TcV, showed the highest degree of resistance to both drugs [75].

Early studies reported considerable anti-*T. cruzi* activity of POSA in vitro and in murine infections [78,79] and that POSA in combination with BZN promoted parasitological cure in mice [80,81]. When more powerful in vivo models of testing were used it became clear that the antiparasitic activity of POSA was inferior to that of BZN and that the triazole only temporarily reduces parasitemia without achieving a cure [82,83,84].

POSA and ravuconazole were the first drugs to enter clinical trials for Chagas disease. Results from these trials indicate clinical failure of the ergosterol biosynthesis inhibitors and their inferior performance in relation to BZN [85,86]. The in vitro and in vivo observations mentioned above may partly explain the clinical findings.

To establish whether there were statistically significant differences in susceptibility to BZN among DTUs, a systematic review was undertaken [71]. A total of 60 articles covering 189 assays were included in this analysis. Of these, 97 assays were performed on epimastigotes, 51 on trypomastigotes, and 60 on amastigotes. The reviewed articles encompassed 59 *T. cruzi* strains belonging to six DTUs (40 TcI, 6 TcII, 3 TcIII, 1 TcIV, 5 TcV, and TcVI strains). The strains were originated from seven Latin American countries, with Brazil contributing the most, followed by Colombia and Mexico. Based on the selected studies, the authors performed a comprehensive meta-analysis of BZN IC_50_ or LC_50_ mean values for different parasite stages and DTUs and concluded that, due to several limitations, it was not possible to attribute specific susceptibility or resistance characteristics to BZN to each DTU.

Considering these observations and the urgent need for novel drugs and treatments for Chagas disease, a committee issued recommendations: (i) drug discovery efforts for Chagas disease should consider the diversity of *T. cruzi* strains; (ii) a panel of strains representing the diverse lineages should be used for in vitro primary screens and lead compounds selection; (iii) screening should be performed on intracellular amastigotes [87]. For details regarding promising approaches for Chagas disease treatments, including drug repurposing and combination therapy, refer to [88].

Many studies for in vivo drug screening use mouse models. Although the approaches for in vivo screening have improved in recent years, using highly sensitive in vivo imaging [89], this system is infeasible for the scrutiny of large compound libraries or the analysis of the behavior of multiple *T. cruzi* strains.

In addition to natural drug resistance, variation in susceptibility to BZN and NFX can be ascribed to the emergence of drug resistance within a *T. cruzi* population. Drug-resistant *T. cruzi* isolates can be selected in the laboratory by BZN pressure [66,90,91,92]. Preliminary evidence suggests that this phenomenon may also occur in vivo [74]. The main mechanisms associated with natural and acquired drug resistance include drug inactivation, modification of the drug target, prevention of drug uptake and active drug efflux [93]. To gain insights into mechanisms that lead to resistance against nitroheterocylic drugs, *T. cruzi* drug-resistant clones obtained by in vitro drug-selection were investigated [66,94,95,96]. These studies identified the gene encoding the TcNTR enzyme, responsible for activating nitroheterocyclic pro-drugs [66,67], as an important player in acquired resistance to BZN and NFX. Furthermore, genome-wide accumulation of mutations was observed in the resistant parasites, revealing the highly mutagenic activity of BZN metabolites in *T. cruzi* [96].

ATP-binding cassette (ABC) transporters, involved in the translocation of a variety of molecules across cellular membranes, have been implicated in drug resistance in protozoan parasites since these transporters could mediate the efflux of drugs [97]. We reported that the single copy ABCG transporter gene, named *TcABCG1*, is over-expressed in *T. cruzi* strains considered naturally resistant to BZN and exhibits single nucleotide polymorphisms (SNPs) in relation to the gene of BZN-susceptible strains [98,99]. The sequence of *TcABCG1* gene of fourteen *T. cruzi* strains, with diverse degrees of BZN susceptibilities and belonging to the six DTUs and Tcbat, showed DTU-specific SNPs that result in amino acid changes. However, so far, no direct correlation between amino acid changes and BZN resistance phenotype was found [99].

Transiently dormant, non-replicating intracellular amastigotes may represent another form of drug resistance in *T. cruzi* [42]. However, the transition into dormancy occurs at a very low frequency and the dormant amastigotes return to an active dividing state that is sensitive to drugs [42].

## 4. Association of the DTUs with Mammalian Reservoirs

Parasitism is an ecological relationship involving three interconnected subsystems: the parasite, the host, and the environment. This relationship evolves over thousands of years through adaptive and co-evolutionary processes. Throughout this evolutionary process, various animal species can become hosts for parasites. In most cases, this relationship does not result in disease and the host is considered a reservoir for the parasite. However, in some cases, the parasite can cause serious damage to the host, resulting in the development of a disease [100]. In the upcoming chapters, we will review records concerning the association of the DTUs with mammalian reservoirs and humans.

American trypanosomiasis was originally an enzootic disease, affecting exclusively wild mammals and being transmitted by triatomines with wild habits [101]. In some regions of the Americas, this pattern persists. However, in other areas, the parasite was introduced into artificial niches, leading to infections in humans and susceptible domestic animals, with transmission occurring through domiciled triatomines.

*T. cruzi* is a common parasite of small mammals, particularly opossums and nest-building rodents, widespread in the Americas, from the southern USA to Argentina. The most likely route of transmission among small mammals is believed to occur orally, when the animal ingests an infected bug or licks triatomine fecal deposits from its fur [102]. The demonstration of *T. cruzi* development within the anal glands of opossums [103] suggested that the parasite was able to penetrate the oral mucosa and travel through the bloodstream to these glands. Therefore, it was proposed that *T. cruzi* could be transmitted directly between opossums via their anal gland secretions, as well as by blood-sucking insects [104].

This set of evidence led Schofield [104] to propose a brilliant theory that the earliest forms of *T. cruzi* would have been associated with marsupial opossums in the southern supercontinent Gondwanaland during the early Tertiary period, about 65 mya. After the separation of the continents about 40 mya, opossums would have migrated to South America. In the Plio-Pleistocene epoch, when North and South America rejoined, South American marsupials migrated into North America. During this period, blood-sucking Triatominae did not yet exist, so trypanosomes would have been transmitted directly between opossums via their anal gland secretions and/or urine. By the late Tertiary or early Pleistocene, approximately 2–5 mya, opossums would have become widespread throughout South America, along with other nest-building vertebrates such armadillos and cricetid rodents. Reduviid predators would have also been present in the ecosystem, feeding on the blood of opossums infected with *T. cruzi*, thereby spreading trypanosomes to new hosts. In this scenario, *T. cruzi* would have been subjected to high selective pressure, leading to the emergence of parasite “variants” that can be distinguished today by a series of genetic markers [104].

The dating for the evolutionary history of *T. cruzi* DTUs is supported by phylogenetic analyses of nucleotide sequences from several nuclear genes and one mitochondrial region [36]. The data indicate that the current lineages must have diverged within the last 3 mya, and that the major hybridization event leading to hybrid lineages TcV and TcVI occurred less than 1 mya, well before the contact of *T. cruzi* with humans in South America.

The sylvatic cycle of *T. cruzi* is constituted of ecological units, in which wild mammals harboring the parasite serve as a food source for wild triatomines. These triatomines become infected and transmit the parasite to other susceptible mammals, ensuring regular and continuous transmission of the parasite regardless of the human presence. This situation can persist indefinitely as long as the ecological balance is maintained [101]. However, the natural foci of *T. cruzi* represent a potential epidemiological risk for the establishment of Chagas disease in the domestic cycle. In this way, the study of the transmission cycle among wild animals has been of interest to researchers for several decades, and the advances in molecular techniques for *T. cruzi* lineage characterization have driven studies on the associations between mammalian species and *T. cruzi* genotypes.

Because of the high genetic diversity of *T. cruzi*, this parasite is capable of infecting more than 100 species of wild mammals, including opossums, armadillos, bats, carnivores, rodents, and primates. Several articles and reviews describe the distribution of *T. cruzi* DTUs in wild reservoirs. References of such studies can be found in recent reviews [9,105,106,107].

**Figure 2:**
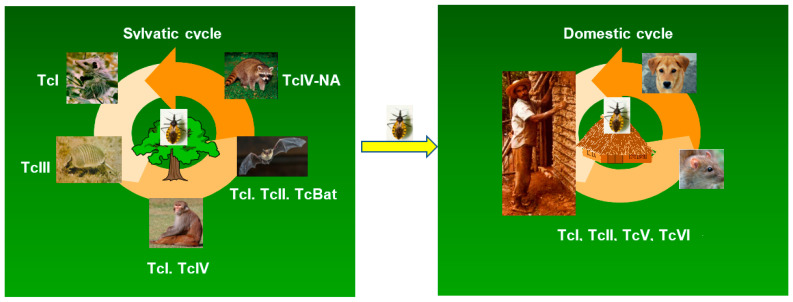
Distribution of *T. cruzi* DTUs in the sylvatic and domestic cycles of transmission. The figure illustrates the consensus of the preferential, but not exclusive, association of DTUs to certain species/orders of wild mammals. In the domestic cycle, the DTUs prevalent in humans are indicated.

In this review, the available information regarding the association of the dominant DTUs in wild mammal species will be summarized. Figure 2 illustrates the text presented below.

In most studies, the genotyping of *T. cruzi* populations was performed after isolation and amplification of the parasites in culture, employing PCR schemes targeted to a set of molecular markers [24]. This approach has disadvantages discussed by others [108,109]. The main drawback associated with parasite isolation is the possible selection of subpopulations best adapted to grow in axenic medium. Furthermore, PCR genotyping methods have limited sensitivity that determines the identification of the prevalent genotype and ignores the possible multiclonality of the infections. Therefore, the development of more sensitive methods to characterize the parasite subpopulations may modify the panorama presented below.

As mentioned earlier, in the remarkable work of Brenière and collaborators, data from articles published until April 2016 concerning the distribution of DTUs in humans, vectors and mammalian reservoirs from the southern USA to Argentina were surveyed [30]. To support the discussion that follows, we compiled Table 1 from data obtained in this inventory [30]. The Table describes the distribution of DTUs in wild mammals categorized into six orders. For a quantitative analysis, we arbitrarily selected only samples with ten or more reported isolates. The data clearly show that DTU TcI predominates in the wild environment, being found in five mammalian orders, while DTUs TcV and TcVI are poorly represented and have been described in representatives of the Rodentia order.

### 4.1. DTU TcI and Opossums

Many studies unequivocally attest that TcI is the major DTU infecting *Didelphis* species (Order Didelphimorphia) from Argentina, Brazil, Chile, Colombia, Mexico, the southern USA, and Venezuela. Interestingly, no other numerically expressive DTU was found in this mammalian reservoir (Table 1). This suggests that at least some *Didelphis* species can control infection by strains of other DTUs. In fact, *D. marsupialis* infected with the Y strain (DTU TcII) presents a short period of patent parasitemia, followed by rare positive blood examinations, along with no parasitism in the anal gland. Conversely, infections of *D. marsupialis* with the G strain (DTU TcI) give rise to high parasitemia and heavily parasitized anal glands [110]. The mechanisms by which opossums would eliminate or reduce infection with TcII strains remain unknown.

### 4.2. DTU TcIII and Armadillos

Infection of armadillos (Order Cingulata) with DTU TcIII has been consistently reported in focal transmission cycles in Argentina, Brazil, Colombia, Mexico, Paraguay, and Venezuela. As shown in Table 1, DTU TcIII has apparently not been found in other mammalian reservoirs, except for a low number of isolates from rodents [30].

Important insights into the transmission dynamics of Chagas disease have been obtained from the Gran Chaco region, a hot and semiarid area divided among eastern Bolivia, western Paraguay, and northern Argentina. This region appears to be the distribution center of *Triatoma infestans*, the main domestic vector of Chagas disease in South America. In the Gran Chaco, TcI and TcIII are the major DTUs found, respectively, in opossums and various species of armadillos, while TcV and TcVI are the most prevalent DTUs in domestic hosts [111,112]. This shows the clear separation of *T. cruzi* DTUs in the sylvatic and domestic transmission cycles, although these genotypes coexist in the same region.

### 4.3. DTU TcBat and Bats

As the name implies, the DTU TcBat was initially described in species of bats, predominantly of the genus *Myotis* (Order Chiroptera), captured in Central and Southeast Brazil [23]. In Brazilian Amazonia, other bat species harbor DTU TcI. Phylogenetic analyses have demonstrated that the bat isolates fall in different *T. cruzi* clades and that TcBat is closer to TcI than to other DTUs [23,25]. TcBat has been reported in bats from Panama [113], Colombia [114], and Ecuador [115]. Interestingly, although TcBat DNA has been detected in different wild triatomine species [115,116], this lineage has not been described in any other mammalian reservoir. Furthermore, reports in humans are extremely rare, with TcBat DNA being found in one child in Colombia [114] and in the hearts of mummies from Chile [117].

### 4.4. DTU TcIV-NA and Racoons

The inspection of Table 1 reveals the distribution of TcIV in reservoirs belonging to the Carnivora and Primata orders, with the former having a significantly larger number of samples.

Phylogenetic analyses of *T. cruzi* populations within this DTU clearly demonstrate the separation of South American and North American TcIV lineages (see references in [118]). The divergence between South America TcIV (TcIV-SA) and North America TcIV (TcIV-NA) is consistent with their preferred hosts and geographical distances. TcIV-SA was primarily identified in wild primates, inhabiting arboreal ecotopes in Brazilian Amazonia [119], whereas TcIV-NA has been found in terrestrial raccoons (Carnivora order) in the USA [120,121,122].

Most sampled raccoons (*Procyon lotor*) from the eastern USA were infected with TcIV-NA, whereas *Didelphis* spp. from the same region were almost exclusively infected with DTU TcI, indicating specific host–DTU associations. In fact, attempts to experimentally inoculate opossums with a TcIV-NA isolate obtained from raccoon did not result in infection [123]. Both TcI and TcIV-NA lineages were described in *Triatoma* spp. from Georgia, Florida, Texas, and southern Arizona [118].

### 4.5. DTUs and Non-Human Primates

Available reports show that there is no specific non-human primate-DTU association. In Brazilian Amazonia, these reservoirs were infected with TcI and TcIV-SA parasites, and, apparently, could be transmitted by *Rhodnius* species [119]. Interestingly, neither TcIV-SA nor TcIV-NA were detected in any of the 17 non-human primates captured in southeastern Mexico. Instead, these reservoirs harbored TcI, TcII, TcIII, TcV, and TcVI parasites. A remarkable situation is the association of TcII with the non-human primate golden lion tamarins (*Leontopithecus* genus) captured in the Atlantic coastal rainforest of Brazil [124]. This reservoir maintains a stable TcII infection with high parasitemia [125].

### 4.6. DTUs in the Domestic Transmission Cycle

In contrast to the well-established associations of TcI, TcIII, TcBat, and TcIV-NA with *Didelphis* opossums, armadillos, bats, and raccoons, respectively (Figure 2), there are scarce studies, and often restricted to a few wild foci, for the other DTUs. This is the case for the TcV and TcVI DTUs, which are rarely found in the sylvatic cycle (Table 1), but they are prevalent in the domestic transmission cycle in several South American countries, as will be reported below.

The domestic cycle is established when triatomine species invade and colonize human dwellings, following anthropic environmental changes and damage to triatomine natural biotopes. As a result, humans and domestic animals become part of the epidemiological chain of Chagas disease, with the potential for *T. cruzi* exchange between the sylvatic and domestic cycles (Figure 2). Among the triatomine species adapted to live in human dwellings or peridomiciles, *Triatoma infestans*, *Rhodnius prolixus*, *Panstrongylus megistus*, *Triatoma dimidiata*, and *Triatoma brasiliensis* are included. These vector species have a differential geographical distribution [126]. Some reports indicate a possible vector–DTU-specific association [9,108]. In the Gran Chaco region, TcV, TcVI, and TcII have been found in domestic dogs and cats, as well as in domestic and peridomestic triatomines (*T. infestans* and *T. sordida*). In fact, dogs play an important role as domestic reservoir of *T. cruzi* in many regions of the Americas [127]. In rural areas, dogs may roam into the wild, become infected, and introduce the parasites into households. Both synanthropic rodents and dogs have high reservoir host competence, contributing to the amplification of *T. cruzi* and serving as a blood source for domiciled triatomine species (Figure 2). As will be described below, the DTUs TcI, TcII, TcV, and TcVI are predominant in the domestic cycle.

## 5. DTUs and Human Chagas Disease

Here we will review data regarding the DTUs prevalent in patients in the chronic phase of Chagas disease. It is important to note that in areas of high endemicity inhabitants can be repeatedly infected through contact with various triatomines, which, in turn, may have fed on several human hosts or mammalian reservoirs. As a result, mixed infections with distinct parasite populations may be expected and, accordingly, the ability to detect the variety of the infecting population is limited by the resolution of the genotyping approach. In most studies the characterization of *T. cruzi* DTUs in patients was determined by PCR schemes, applied to parasites obtained from peripheral blood, which had previously been amplified in culture. In a few cases, DTU genotyping was performed directly in blood samples or tissue biopsies. The limitations of these approaches have been discussed above.

### 5.1. Geographical Distribution of DTUs in Patients

In 2022, Velásquez-Ortiz and colleagues performed a systematic review of the literature from the last 20 years and updated knowledge on the distribution of DTUs in the Americas [128]. Carefully compiled maps indicate that DTUs are widespread across the continent. The geographical distribution of the DTUs most frequently found in patients from Chagas disease endemic countries has been addressed in several review articles [5,8,30,108,129]. In a recent review by Magalhães et al. [5], we report an updated landscape of the geographical distribution of *T. cruzi* DTUs in humans, an illustrative map, and recent references. This information will be summarized and discussed below.

DTU TcI predominates in autochthonous human cases from the USA, Mexico, and Central American countries. In South America there is greater DTU diversity. TcI is responsible for most human infections in the Amazon basin, the northern countries of South America (Venezuela and Colombia), as well as in patients from the southern Cone countries. Given the considerable genetic diversity of DTU TcI, it is likely that different TcI subpopulations circulate in these regions. In Bolivia, in addition to TcI, the DTUs TcII, TcV, and TcVI also infect humans. In peripheral blood samples of Chilean patients TcII, TcV, and TcVI were detected in single or mixed infections. Both TcV and TcVI are the main DTUs in the domestic cycle in Argentina. In the southern and southeastern regions of Brazil, TcII is reported more frequently in human infections, whereas TcIV predominates in patients in the northern region. Interestingly, the DTU TcIV is the second most common cause of Chagas disease in Venezuela and Colombia. *T. cruzi* DTUs were also characterized in Bolivian migrants attending hospitals in Spain and TcV was the most common DTU found [130,131].

The differential geographic distribution of DTUs in patients reflects host–pathogen interactions, which include the genetic characteristics of both players. This anticipates regional variations in the host antibody response, which, in turn, would impact the performance of several commercial serological diagnostic tests. Indeed, considerable variability in the sensitivity of current diagnostic tests has been reported in Latin American countries [8,62]. Therefore, it is recommended that the performance of commercial serological tests be evaluated in countries endemic for Chagas disease, and that the validation of new diagnostic tests should include sera of patients from endemic countries.

### 5.2. Clinical Implications of T. cruzi DTUs

One of the primary objectives in classifying *T. cruzi* strains into major lineages or DTUs has always been to explore their potential implications in determining the various clinical manifestations of Chagas disease. Despite some progress, our ability to draw definitive conclusions remains somewhat constrained. While the initial hope of finding different DTUs responsible for distinct clinical forms of the disease did not materialize, some DTUs seem to contribute to increase the risk of certain morbidities in comparison to others. On the other hand, since host–pathogen interactions are complex, with several unknown aspects influencing the fate of infections, the interplay between the pathogen and the host’s immune response must be critical in determining disease evolution.

From a global perspective, it is interesting to note that the geographical distribution of the major *T. cruzi* lineages, summarized above, closely mirrors the severity gradient of Chagas disease within this range. The alignment between the distribution of *T. cruzi* DTUs and disease severity has led to a general, though not absolute, association of TcI with acute and/or milder chronic cardiac disease and TcII, TcV, and TcVI with more severe chronic cardiac or digestive disease. TcIII, TcIV, and especially TcBat are rarely associated with human infections (revised by [132]). Table 2 presents an overview of the key findings, associating DTUs and the clinical aspects of Chagas disease obtained thus far. Additional and updated details on the clinical implications of each DTU can be found below.

#### 5.2.1. TcI

As previously highlighted, TcI stands as the most widespread DTU, prevailing in both sylvatic and/or domestic cycles in Mexico, Central America, and countries within the Amazon region (as reviewed by [30,133]). The specific role of TcI in determining the clinical manifestations of Chagas disease is not completely understood, but the rarity of megasyndromes (megaesophags and megacolon) in regions where TcI prevails has been attributed to its predominance. On other hand, the occurrence of cardiac disease associated with TcI has been extensively documented in some countries above the Amazon region, particularly in Colombia and Venezuela [134]. Studies involving the typing of *T. cruzi* directly in heart samples of cardiac Chagasic patients who underwent heart transplantation have revealed that mixed infection with both TcI and TcII are more common than initially anticipated in these countries. This discovery has led to the hypothesis that the occurrence of cardiac manifestations in patients infected with TcI could, at least in part, be explained by the presence of mixed infections [135,136]. However, instances of disease and death due to TcI, even in regions where TcII is scarce or absent, indicate that pathogenicity is an inherent characteristic of TcI strains [137]. Furthermore, Chagas cardiomyopathy manifestations seems to be more strongly correlated with TcI than with TcII, at least in Colombia [134].

Curiously, in contrast to the endemic regions located above the Amazon rainforest, chronic human infections by TcI strains appear to be rare and typically asymptomatic in Southern Cone countries, where TcII, TcV, and TcVI prevail. In these regions, TcI is only occasionally associated with severe acute cases, with most of them resulting from oral infection outbreaks and the incursion of sylvatic rodents into houses. TcI has also been detected in association with reactivated cases of Chagas disease in severely immunocompromised individuals, particularly in cases of central nervous system complications and meningoencephalitis [138]. The explanations for why TcI is associated with clinical cases in some endemic regions and not in others are still to be clarified.

In this context, substantial genetic heterogeneity has been attributed to TcI, as demonstrated by various studies [139,140,141,142,143]. This genetic diversity is evident in localized studies conducted in different regions, including Mexico [144], Colombia [139], Venezuela, and Bolivia [142]. Based on these studies, attempts to subdivide TcI strains into clinic relevant groups have been reported. The initial results seemed promising, with some haplotypes, particularly TcIDOM (formely TcIa), associated with the domestic/peridomestic transmission cycle and human infections in regions such as Colombia [145,146] and Venezuela [142]. However, further studies conducted in other endemic areas have challenged the confirmation of this association [147,148].

#### 5.2.2. TcII, TcV, and TcVI

TcII, along with its relatively more recent derivatives, the hybrid DTUs TcV and TcVI, are unequivocally associated with the most severe chronic cases of Chagas disease and are widespread in central, southern, and southeastern South America. These DTUs are closely related genetically, and distinguishing between them can be a challenging task, depending on the parasite load and the genotyping method employed. Therefore, TcII, TcV, and TcVI are sometimes collectively referred to as “non-TcI” [149].

TcII is the predominant agent of Chagas disease in the Southern Cone region of South America, particularly in areas where *Triatoma infestans* is or was the main domestic vector. In these regions, TcII is responsible for more than 90% of chronic infections, manifesting in cardiac, cardiodigestive, or indeterminate forms [150]. Notably, this DTU is the primary cause of severe cases of Chagas disease in South America, especially in central and southeastern Brazil. In these areas, the prevalence of patients with the cardiac clinical form can exceed 40% among patients in the chronic phase of the disease [150,151,152]. This contrasts with the average of 10–15% observed in other endemic areas where TcII is rare or absent [153]. In southeastern Brazil, both the cardiac and digestive tissues lesions are predominantly associated with TcII strains [151,154]. Furthermore, TcII has been linked to severe chronic Chagas cardiomyopathy and with patients who experience heart failure or sudden cardiac arrest in these regions [155].

TcV plays a significant role in human infections, especially in the Gran Chaco region and neighboring countries, including Bolivia, Chile, northern Argentina, and southern Brazil [156,157,158]. For example, megasyndromes in Bolivia are predominantly associated with TcV and, to a lesser extent, with TcII [156]. Moreover, in this region, which has one of highest incidences of Chagas disease nowadays, most cardiac patients seem to present mild heart disease and do not progress to a severe heart condition when compared to infected patients in southeastern Brazil and southern Argentina [159]. At least in part, these specific characteristics of Chagas disease in the Gran Chaco region are potentially associated with TcV, the prevalent *T. cruzi* DTU in this area. Interestingly, this may also be associated with the relatively widespread belief in Bolivia that if infected patients avoid unnecessary physical exertion and maintain a peaceful life, Chagas heart disease is not a serious illness [160].

Similarly, in a comprehensive recent study conducted in Argentina, TcV was identified in 47.7% of the infected population. However, the probability of finding TcV in patients who have not developed the disease after 20 years of infection was significantly higher than in patients with chronic Chagas cardiomyopathy [161], reinforcing the idea that TcV is also more associated with milder heart disease, at least, in Argentina.

But, without a doubt, the most intriguing characteristic of TcV is its potential association with congenital transmission of Chagas disease in Chile, Bolivia, Paraguay, Uruguay, and southern region of Brazil. The frequency of congenital infection in all these areas, where TcV predominates, is much higher, ranging from 4% to 6%, compared to relatively rare cases, 1% or less, in areas where TcII and TcI circulate [162]. Whether these correlations result from the intrinsic biological characteristics of this DTU, parasite load, or merely reflect the local abundance of this lineages has been questioned. However, so far, the data available for investigating the direct impact of DTUs on congenital transmission remains limited [163,164,165].

TcVI is highly prevalent in southern Argentina, accounting for 66% of identified cases [161]. It is the leading cause of severe Chagas heart disease in Argentina and the second leading cause in Brazil. Additionally, TcVI, along with TcV, has been associated with the digestive form of the disease in Argentina [166]. There is also evidence that this DTU may increase the risk of congenital transmission of Chagas disease [161]. However, due to challenges some groups face in differentiating TcVI from TcII and TcV, the precise contribution of this DTU in determining clinical aspects of Chagas disease remains to be fully understood.

#### 5.2.3. TcIII, TcIV, and TcBat

TcIII and TcIV are primarily associated with the sylvatic transmission cycle. They have a wide geographical distribution spanning from the southern USA to Argentina, with relatively high prevalence in the Amazon region. However, determining the exact distribution and phylogeography of TcIII and TcIV is challenging due to the limitations of several genotyping methods used in the literature, which often cannot differentiate between these DTUs.

TcIII is rarely associated with human infections, and most of these cases have been reported in the Amazon region, often linked to acute cases. The first clear evidence of the association of TcIII and human infection is just over a decade old, during an investigation of an acute Chagas disease outbreak in Brazilian Western Amazonia [167]. However, this possibility had already been suggested, as TcIII had been detected in domestic dogs in Paraguay and northern Brazil, as well as in peridomestic triatomines in northern and southern Brazil [119,157,168]. Furthermore, outbreaks of acute Chagas disease caused by both TcI and Z3 (TcIII or TcIV) had already been reported [169]. In relation to the chronic infections, TcIII has been detected in five chronic infected patients in Colombia presenting cardiomyopathy, but in mixed infection with TcV [134].

In addition to TcBat, TcIV remains one the least characterized DTU. TcIV is rarely associated with human Chagas disease outside the Amazon basin. However, in the western Brazilian Amazon, this DTU is responsible for most of the acute human infections occurring in Chagas disease outbreaks [170,171]. Notably, TcIV was responsible for the first recorded putative outbreak of orally transmitted simultaneous acute cases of Chagas disease in Canudos, Belém, Pará State, Brazil [172]. Subsequently, several cases of oral transmission were confirmed in the Amazonia basin, which is nowadays considered an endemic area for this mode of transmission of Chagas disease [169].

The overlapping high prevalence of TcIV with TcIII and TcI in the Amazon region has led to the hypothesis that these DTUs are particularly associated with severe acute cases of Chagas disease caused by contaminated foods and beverages. In addition to acute outbreaks, TcIV has also been detected in chronic infected humans in Guatemala, Peru [173] and Venezuela [7,174]. In fact, different studies point out that TcIV is the secondary cause of Chagas disease in Venezuela [7,174]. For instance, among 95 isolates genotyped from human disease cases in Venezuela, 79% belonged to TcI and 21% belonged to TcIV, with most of them being acute or asymptomatic cases [174].

TcBat is the most recently defined DTU and unquestionably one of the least studied among the seven major *T. cruzi* lineages. Through epidemiological surveillance, TcBat DNA was reported in a 5-year-old female living in a forest area in northwestern Colombia [114]. Molecular analyses confirmed a mixed infection involving both TcI and TcBat genotypes in this child. TcBat DNA was also found in the hearts of Chilean mummies [117]. Although one TcBat strain has demonstrated the ability to invade mammalian cells, it was not associated with tissue damage or any pathological changes in the child, as evidenced by the absence of ECG alterations [114]. Due to the genetic similarity between TcBat and TcI, and the requirement for specific molecular markers, which are often not included in the majority of current DTU genotyping methods, our comprehension of the precise role of TcBat in human Chagas disease remains very limited.

## 6. DTUs and the Etiological Treatment of Chagas Disease

The major goal of the etiological treatment of Chagas disease is to eliminate the parasite from the individual. This aims to reduce the risk of developing symptomatic pathologies and to prevent parasite transmission through vectorial, transfusional, and congenital routes.

Considering the differential geographic distribution of DTUs prevalent in Chagas disease patients, we sought information on possible geographic variation in the response to treatment with the drugs currently in use. An extensive study conducted by Médecins Sans Frontières evaluated BZN efficacy in children from Honduras, Guatemala, and Bolivia [175]. Seroconversion occurred 18 months post-treatment in children from Honduras and Guatemala and much later, up to 60 months, in children from Bolivia. The variability in the apparent treatment outcome was attributed mostly to differences in the parasite populations circulating in these countries [175]. In fact, and according to the available data, TcI predominates in patients of Central America, while TcII, TcV, and TcVI, predominate in patients in Bolivia [8,128].

The BENEFIT (Benznidazole Evaluation for Interrupting Trypanosomiasis) trial tested the hypothesis that trypanocidal therapy is beneficial for patients with chronic Chagas heart disease [176]. Although the general conclusion of the study was that BZN failed to halt disease progression, clear differences in response among patients were observed. That is, the reduction in the parasite load, assessed by the conversion rate to negative PCR results, was more pronounced in patients from Brazil than in patients from Colombia and El Salvador, while patients from Argentina and Bolivia presented intermediate values [176]. The debate remains whether the lack of BZN efficacy reflects differences between the infecting strains and/or the fact that patients already had established heart failure manifestations. Unfortunately, in the BENEFIT trial there is no information regarding the *T. cruzi* genotype found in the patients. Notably, a non-randomized study of BZN treatment showed the beneficial effect of the drug in preventing disease progression in patients from Argentina in whom cardiac damage had not yet occurred [177].

Nevertheless, the regional differences in the effect of BZN in reducing the parasite load cannot be attributed exclusively to the genotype of the infecting strain, since, as discussed above, BZN and NFX have a wide range of activities against strains of different DTUs, and even against representatives of the same DTU. Therefore, the genetic characteristics of the infecting strain and the host, the host’s immune response, as well as other unknown factors must influence the treatment outcome.

Currently, there is a lack of satisfactory methods to measure therapeutic responses in the chronic phase and to certify the cure. In the first clinical trials, the seroconversion of conventional serological tests, which can occur up to 10 years after treatment, defined cure [178]. More recently, the amplification of *T. cruzi* DNA, using qPCR, is the main method for evaluating responses to treatment in a short period of time. However, the sensitivity of this approach may be insufficient for diagnosing cure. Therefore, biomarkers of treatment response as well as disease progression are an important medical need for Chagas disease. Research on both parasite- and host-derived markers is ongoing (reviewed by [179,180]). Recently, a multiplex immunoassay disclosed the steady decline in antibodies to *T. cruzi* specific antigens in BZN-treated mice, which correlated with highly sensitive bioluminescence imaging [181]. The availability of different types of biomarkers deserves evaluation in longitudinal monitoring of drug-treated patients and in clinical trials.

## 7. Is *T. cruzi* a Single Species?

With the information presented above, we now discuss the question posed by some authors, whether *T. cruzi* is a single species or if *T. cruzi* may be considered a group of related species.

The contemporary biological species concept integrates the notion that a species is not only a reproductive community but also an ecological and genetic unit. Obviously, for essentially clonal species like *T. cruzi* or *Leishmania*, defining them in reproductive terms is not feasible. Consequently, the concept of genetic unity, denoting shared genetic markers or features, becomes crucial. In this context, the consideration emerges about the acceptable threshold of intraspecific genomic variability before contemplating the division of a species into distinct entities [182].

Historically, *T. cruzi* has been regarded as a single species despite its substantial genetic diversity, demonstrated by the distinct DTUs. However, a recurring question is whether *T. cruzi* should be subdivided into subspecies or separate species [183,184]. Advocates for subdivision argue that the genetic, biological, and ecological variations among DTUs are substantial enough to justify classifying them as distinct species [185]. They emphasize that *T. cruzi* exhibits phylogenetic diversity comparable to the genus *Leishmania*, which has over 20 species associated with human leishmaniasis. Despite some criticism regarding the abundance of species in *Leishmania*, the supporters of *T. cruzi* subdivision into species stress that the phylogenetic divergence among *Leishmania* species aligns with lower clades in *T. cruzi* [184]. With the evidence available in the 1990s, the authors argued that varying clinical manifestations associated with different *T. cruzi* DTUs have potential implications for disease progression and treatment response, warranting formal taxonomic subdivision [183,184]. Conversely, skeptics of species splitting assert that the current classification adequately encompasses the intraspecific diversity of *T. cruzi*, and that observed variations can be accommodated within a single species framework [186]. They advise against premature taxonomic changes, citing potential repercussions for research, diagnostic, and public health strategies.

Arguments against subdivision include the presence of hybrid parasites resulting from at least two hybridization events between main *T. cruzi* DTUs (TcII and TcIII and/or TcI and TcII) [32,34,35]. Furthermore, unlike most *Leishmania* species, genetic distances within each *T. cruzi* DTU are not substantially lower than those observed across the entire species, with a significant portion of *T. cruzi* diversity residing within DTUs [186]. Moreover, the association of specific DTUs with the clinical manifestations of Chagas disease and treatment resistance lacks clear demonstration, as we have discussed above. Therefore, in the view of these researchers, with whom we agree, *T. cruzi* should be maintained as a single species both from a reproductive, ecological, and genetic perspective.

## 8. Conclusions

Phylogenies derived from conserved genes, concatenated gene sequences, or complete genome alignments consistently support the division of *T. cruzi* strains into the seven DTUs proposed fifteen years ago. Most DTUs exhibit extensive intragroup genetic diversity, and the relevance of establishing genetic subdivisions within these DTUs has been a subject of debate without reaching a consensus.

Compelling evidence from comparative genomic analyses of strains from different DTUs indicates a sexual cycle in *T. cruzi*, occurring alongside broad clonal expansion. These processes have played a significant role in shaping the genetic diversity of this parasite, with profound epidemiological implications. Genomic data reveals substantial variability between and within DTUs in both copy number and sequence of genes encoding surface proteins. These polymorphisms likely have provided the parasite with mechanisms to evade the host’s immune response, presenting a formidable challenge for the development of vaccines against *T. cruzi*. Simultaneously, the expansion and diversification of surface molecules most probably ensured the parasite’s success in establishing infections in different mammalian hosts and vectors.

The classification of *T. cruzi* strains into DTUs has been pivotal in unraveling ecoepidemiological and pathogenic characteristics of *T. cruzi*. It has brought to light the preferential association of specific DTUs with certain species or orders of mammalian reservoirs and has revealed the differential geographic distribution of DTUs, prevalent in patients from Chagas disease endemic regions. While the initial expectation of distinct DTUs causing specific clinical forms of Chagas disease has not been entirely met, certain DTUs have shown a propensity to increase the risk of specific pathologies. For instance, TcI, the most widespread DTU, has been implicated in cardiac manifestations in some areas, while TcII, TcV, and TcVI are linked to severe chronic cardiac or digestive disease. However, it is important to consider that host–pathogen interactions are multifaceted with several factors influencing the outcome of the infection, including the host’s immune response and the strategies used by the parasite to evade it. These factors are intimately connected to the genetic characteristics of both actors.

The genetic diversity of *T. cruzi* populations also creates major obstacles to the development of novel therapies for Chagas disease. In vitro studies have shown no clear correlation between susceptibility to BZN and NFX and categorization into DTUs. The reasons for therapeutic failures of these drugs in the chronic phase of the disease are not completely understood, but they are likely not solely determined by the nature of the infecting DTU.

In conclusion, the study of *T. cruzi*’s genetic diversity and its classification into DTUs has provided crucial insights into the ecoepidemiology and pathogenicity of this parasite. It has also shed light on the complex interplay between host and pathogen and the genetic factors influencing disease outcomes. The intricate web of genetic variations within *T. cruzi* populations presents formidable challenges in the quest for effective vaccines and therapies against Chagas disease.

## Figures and Tables

**Table 1:** Distribution of *T. cruzi* DTUs in wild mammals belonging six orders ^a^.

Mammalian Orders	*T. cruzi* DTU							
	TcI	TcII	TcIII	TcIV	TcV	TcVI	TcBat	Total
Carnivora	46	-	-	36	-	-	-	82
Chiroptera	57	21	-	-	-	-	59	137
Cingulata	-	-	78	-	-	-	-	78
Didelphimorphia	262	-	-	-	-	-	-	262
Primata	43	10	-	10	-	-	-	63
Rodentia	91	37	10	-	24	20	-	182
Total	499	68	88	46	24	20	59	804

^a^ Data compiled from [30].

**Table 2:** Clinical aspects of Chagas disease associated with *T. cruzi* DTUs.

DTU	Transmission Cycle	Geographic Distribution	Major Clinical Aspects
TcI	Predominant in sylvatic and/or domestic cycles depending on the endemic region	North and Central Americas and the Amazon region	Associated with cardiac Chagas disease in Venezuela and Colombia. Associated with oral transmission and severe acute cases in Brazil. Associated with neuroencephalitis in immunocompromised patients.
TcII	Predominant in domestic cycle; rare in sylvatic cycles	Southern Cone region of South America	Primary cause of severe cardiac Chagas disease in Brazil. Associated with megaesophagus and megacolon in Brazil.
TcIII	Predominant in sylvatic cycle; rare in domestic cycles	Amazon region	Rarely causes human Chagas disease, mostly in Brazil, associated with oral transmission and acute cases.
TcIV	Predominant in sylvatic cycle; rare in domestic cycles	Amazon region	Few strains have been isolated from chronic infected humans, most of them in Venezuela. Associated with oral transmission and acute cases.
TcV	Predominant in domestic cycle; rare in sylvatic cycles	Bolivia, Chile, northern Argentina, and southern Brazil	Probably associated with milder cardiac chronic Chagas disease. Associated with megaesophagus and megacolon in Bolivia. Possibly associated with a higher risk of congenital transmission.
TcVI	Predominant in domestic cycle; rare in sylvatic cycles	Southern Cone region of South America	Primary cause of severe chronic cardiac Chagas disease in Argentina.
TcBat	Predominant in sylvatic cycle	Panama, central and southeast Brazil, and Colombia	So far, only one isolated case of human infection.

Modified from [132].

## Data Availability

Not applicable.
